# Incarcerated Bochdalek Hernia in Adults

**DOI:** 10.7759/cureus.61422

**Published:** 2024-05-31

**Authors:** Alexis A Granados Flores, Dorian I Arriola Rios, Jose R Gonzalez Soto

**Affiliations:** 1 Surgery, Hospital General Regional No. 66, Juárez, MEX

**Keywords:** incarcerated hernia, diaphragmatic hernia, hemicolectomy, surgery, adult bochdalek hernia

## Abstract

In this case report, the diagnostic challenge and emergency management of a Bochdalek hernia in adults in Mexico are outlined. This case report can help the medical community to consider the clinical presentation in adults and the importance of early diagnosis and management. We present a 57-year-old female patient with a history of arterial hypertension who, following a bout of abdominal pain, was diagnosed with a Bochdalek hernia. Following emergency surgery, there was an increase in intra-abdominal pressure, which was continuously monitored due to the possibility of compartment syndrome, potentially necessitating a second emergency surgery.

## Introduction

Bochdalek first described this diaphragmatic anomaly in 1848. The pathophysiology behind this clinical entity is congenital and results from an improper fusion of the pleuroperitoneal canal. As the right canal closes before the left canal, most Bochdalek hernias (85%) occur on the left side [1]. Among the different types of diaphragmatic hernias, Bochdalek hernia is the most frequently complicated (95%), occurring more commonly on the posterior left side of the diaphragm [2]. Adult Bochdalek hernias are difficult to diagnose. While infants may present with respiratory distress early in life, the most common symptoms in adults are chest and/or abdominal pain (66%) or symptoms of ileus (38%) [3].

Although diaphragmatic hernias are usually diagnosed prenatally or in the immediate postnatal period, in 5% to 25% of cases, the diagnosis may not occur until later and may instead be discovered during routine exams or examinations because of digestive or respiratory issues [4]. Chest X-ray is commonly used for diagnosis; however, initial radiographic findings can be misinterpreted in approximately 25% of cases, necessitating additional diagnostic methods [5].

The most reliable method for identifying diaphragmatic hernias is a CT scan. With a sensitivity and specificity of 14%-82% and 87%, respectively, it is a highly useful tool in cases where initial imaging approaches are unable to confirm a clinical suspicion [6,7]. A CT scan can assess the location and size of the diaphragmatic defect and evaluate various radiological findings of intrathoracic complications of herniated abdominal organs, aiding the surgeon in surgical planning [8].

## Case presentation

A 57-year-old female patient of Mexican nationality, with a history of arterial hypertension diagnosed 10 years ago and managed with calcium antagonists, presented with a one-week history of abdominal pain in the umbilical region, without pain modifiers. The pain was colicky, without radiation, with progressive intensity on a visual analog scale from 4/10 to 10/10 on the day of hospital admission. Additionally, she experienced nausea, vomiting of food content, increased abdominal girth, and absence of bowel movements in the last 72 hours, along with oral intolerance. On physical examination, the patient exhibited neurological deficits characterized by confusion, a Glasgow Coma Scale score of 14, normal temperature, tachycardia, and tachypnea. Oxygen saturation was 92% with supplementary oxygen via nasal cannula at 4 L/minute. Pupils were isochoric, and bowel sounds were present in the left hemithorax. The abdomen was distended and tender to palpation in the mesogastric and epigastric regions, with intact extremities. The laboratory tests performed are described in Table 1.

**Table 1:** Laboratory tests performed.

Parameter	Value	References values	Unit
Prothrombin time (PT)	12.0	11-15	Seconds
INR	1.0	0.8-1.2	-
Activated partial thromboplastin (aPTT)	28.0	25-40	Seconds
Hemoglobin	11.0	12-16	g/dL
Hematocrit	35	37-47	%
White blood cells	8.3	5-10	(x10^3^/μL)
Platelets	224	150-450	(x10^3^/μL)
Glucosa	121.0	60-100	mg/dL
Urea	23.0	17-43	mg/dL
Creatinine	0.68	0.60-1.10	mg/dL
Lactate dehydrogenase	153	140-280	IU/L
Alkaline phosphatase	53	25-100	IU/L
Chloride	109.0	95-105	mEq/L
Potassium	3.4	3.5-5.5	mEq/L
Sodium	135.0	136-145	mEq/L
Calcium	8.7	8.8-10.4	mg/dL
Phosphorus	3.27	2.5-4.5	mg/dL
Magnesium	1.84	1.8-2.5	mmol/L

**Figure 1:**
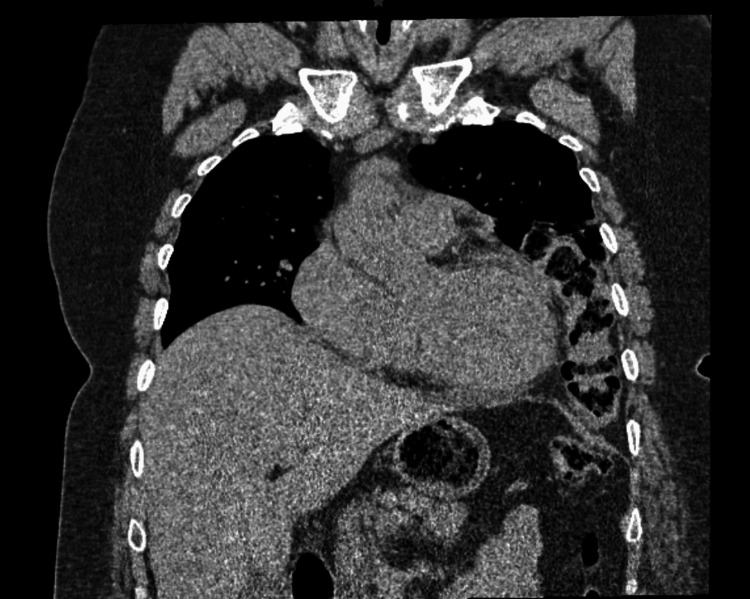
Coronal slice computed tomography (CT) scan: intrathoracic transverse colon. In this figure, the presence of abdominal content in the thoracic cavity can be identified, creating a mass effect and, therefore, restricting the left lung expansion.

**Figure 2:**
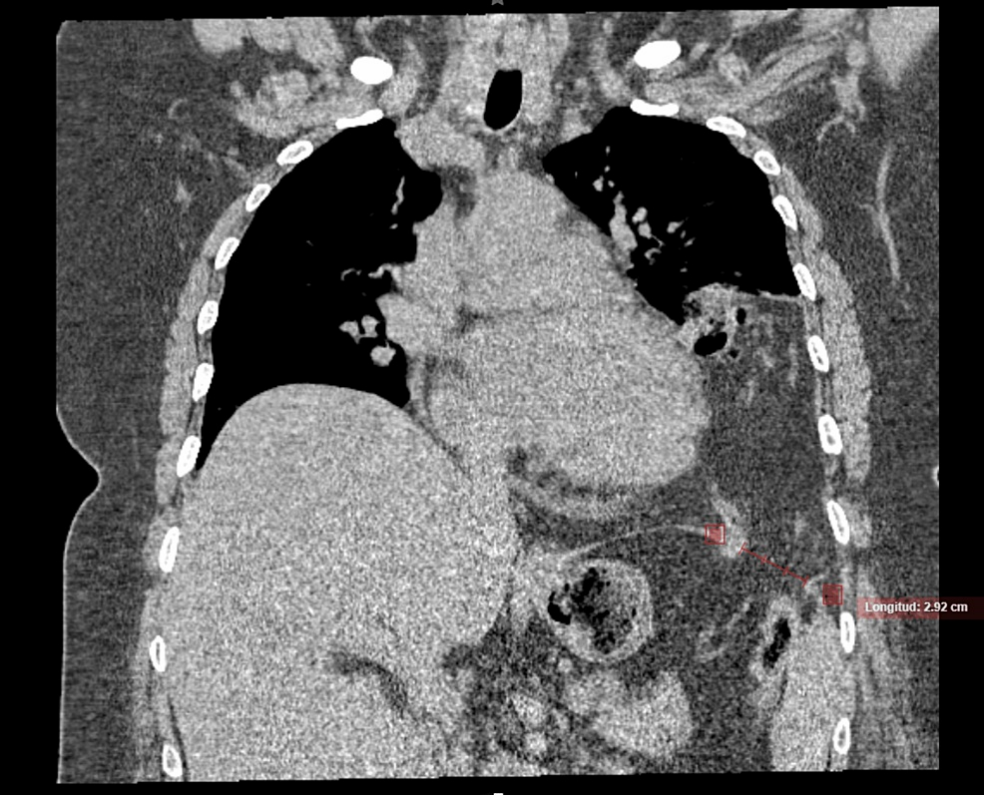
Coronal slice computed tomography (CT) scan: diaphragmatic defect. Diaphragmatic defect with an approximate 2.92 cm diameter.

The patient received decompressive management, including nasogastric tube placement, fasting, and intravenous fluids. Additionally, intravenous analgesics, such as metamizole and ketorolac, were administered. Prophylactic antibiotic therapy, consisting of intravenous metronidazole and ceftriaxone, was initiated to prevent potential bacterial translocation. A contrast-enhanced thoracoabdominal CT was requested, revealing a diaphragmatic hernia with transverse colon and fatty tissue content herniating into the left thoracic cavity, probable torsion with swirling mesenteric vessels sign, dilatation of small and large bowel loops, increased fluid within, and pneumatosis intestinalis at the level of the cecum and ascending colon (Figures 1-2).

Based on clinical and imaging findings, emergency surgery was proposed due to the high risk of intestinal perforation.

Therapeutic intervention

**Figure 3:**
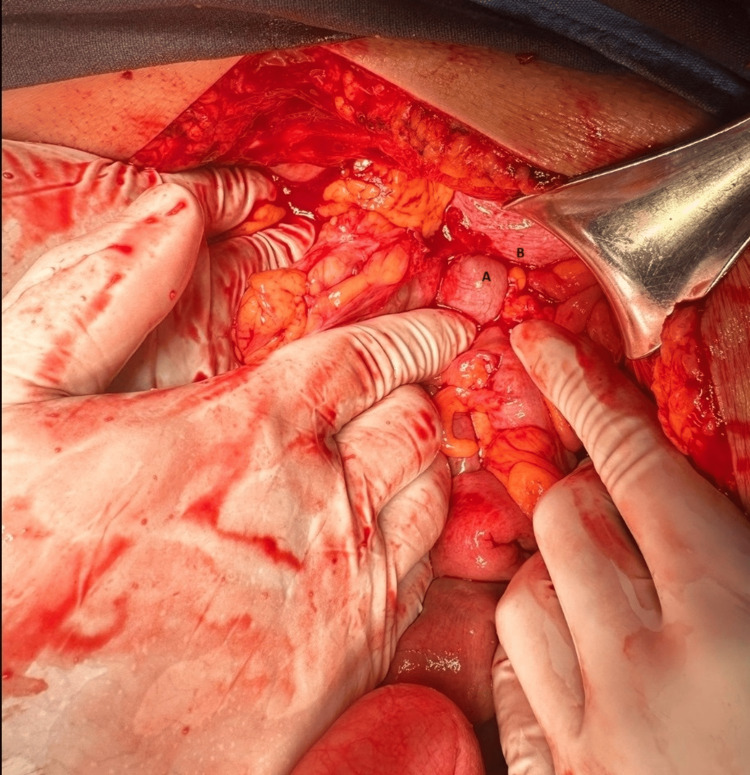
Abdominal view. (A) Transverse colon and (B) diaphragm. During the exploratory laparotomy, we deliberately searched at the level of the diaphragm and identified incarceration of the transverse colon.

**Figure 4:**
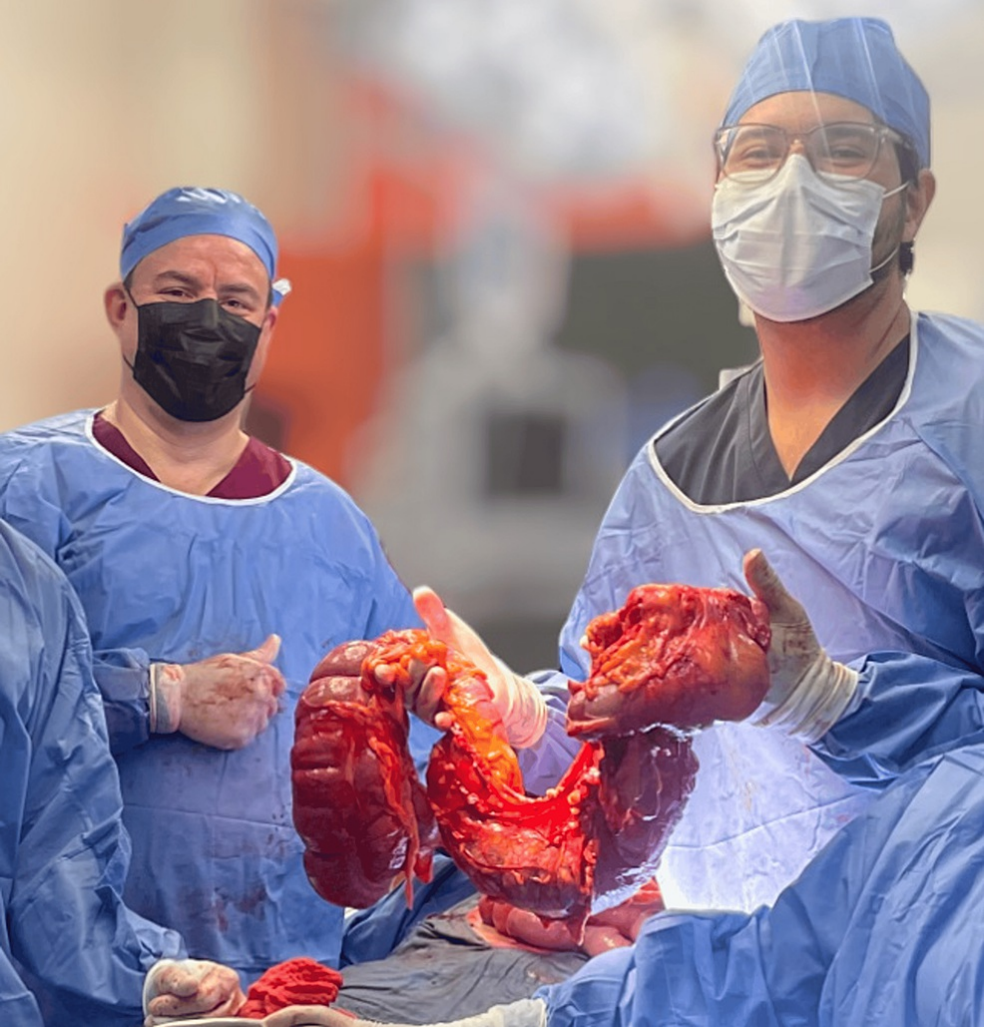
Right colon dimensions. Dr. Sergio Alberto Weckmann Luján and Dr. Alexis Andrei Granados Flores demonstrating the approximately 10 cm diameter of the colon once the extended right hemicolectomy was performed.

The patient underwent exploratory laparotomy with a presurgical diagnosis of intestinal obstruction secondary to diaphragmatic hernia and systemic arterial hypertension. Intraoperative findings included a 4 cm diameter diaphragmatic hernia containing transverse colon without a hernia sac, multiple Zuhlke II and III adhesions in the transverse colon, collapsed left lung, and ascending and transverse colon with 10 cm diameter (Figure 3). As a result of the findings such as colon diameter dimensions compatible with a diagnosis of megacolon, an extended right hemicolectomy with side-lateral intestinal anastomosis was performed using a linear cutter stapler, primary closure of the diaphragmatic defect, placement of a left pleural drainage system and open Penrose drain ¾ inch were performed (Figure 4).

Postoperative management included administering supplemental oxygen via nasal cannula at a rate of 3 L/minute to maintain oxygen saturation goals. The patient continued the same analgesic and antimicrobial therapy regimen. On the first postoperative day, a liquid diet was initiated according to ERAS Society Guidelines, calculated using the Harris Benedict formula. The patient demonstrated adequate tolerance of diet intake throughout the day, experienced sufficient analgesia, and exhibited no fever, normal heart rate, and respiratory rate, as well as intact neurological status and a well-ventilated chest. Within 24 hours post-surgery, 1,000 mL of fluid was obtained from the pleural drainage system, and the surgical wound was well-approximated without bleeding. Additionally, 300 mL of serosanguinous fluid was collected in the Penrose drain, and the urinary index was measured at 1.2 mL/kg/h, with normal bowel sounds.

On the second postoperative day, the patient experienced vomiting during the first intake of a liquid diet, leading to the suspension of the diet. Within 24 hours, 200 mL of fluid was obtained from the pleural drainage system. The patient also exhibited decreased bowel sounds. The progressive increase in abdominal volume necessitated the indirect measurement of intra-abdominal pressure using a central venous pressure measuring device connected to the Foley catheter, revealing grade I intra-abdominal pressure (15 cmH2O). Additionally, there was a reduction in the urinary index to 0.4 mL/kg/h, which we managed by administering 500 mL of Hartmann's solution intravenously to achieve diuresis goals. Consecutive measurements of intra-abdominal pressure were taken every six hours, obtaining pressures of 11.2, 8, and 5 cmH2O, respectively.

On the third postoperative day, 70 mL were obtained from the pleural drainage system. No supplementary oxygen was required to maintain normal saturations. Improvement in abdominal symptoms was noted, and the initiation of a liquid diet was indicated. Intra-abdominal pressure was measured, resulting in 7 mmHg, with subsequent measurements of 5 and 6 mmHg every eight hours. Additionally, the urinary index was recorded at 0.5 mL/kg/h.

On the fourth postoperative day, progression to a pureed diet was achieved without complications. The endopleural tube was removed, followed by radiographic control. The abdomen remained without distension or pain, with normal bowel sounds and intestinal gas evacuation. Intra-abdominal pressure was measured, yielding 5 mmHg, and the urinary index was recorded at 0.8 mL/kg/h.

On the fifth postoperative day, a soft diet was initiated without incident, oxygen saturation remained normal without supplementary oxygen requirement, abdomen continued to be unremarkable, normal bowel sounds, normal evacuation, and the urinary index was 1.0 mL/kg/h.

On the sixth day, the patient was discharged with instructions for surgical wound review and follow-up in the outpatient general surgery clinic in 10 days.

## Discussion

Whether to use an abdominal or thoracic approach to repair a diaphragmatic hernia relies on the surgeon's preferences and level of expertise. However, in the context of a complicated hernia or if the patient exhibits hemodynamic instability, an abdominal approach should be considered [9]. In hemodynamically stable patients, the use of a CT scan is a very important tool that provides us with information to determine the diagnosis and plan the surgery.

Ideally, the method of repair should involve primary closure of the defect using nonabsorbable sutures [10]. Intraperitoneal mesh or grafts may be employed in situations where primary closure without tension is not practical, especially if the hernia defect has an area greater than 20 cm^2^ or a diameter greater than 8 cm [9]. The use of meshes or grafts was not suggested in the particular case of our patient since the defect, which measured around 4 cm, could be satisfactorily repaired with an absorbable suture when the transverse colon was reduced.

It has been shown that the abdominal approach can have direct implications on the percentage of morbidity that patients may experience, considering that an open approach can correspond to 18% compared to what is seen in a laparoscopic approach with 6% [3].

We concur that the best moment for surgery is always in an uncomplicated situation, as seen in previous case reports. As previously mentioned, diaphragmatic hernia diagnosis is often made incidentally during routine studies, as a prophylactic measure against possible future complications every diaphragmatic hernia must be repaired electively once its diagnosis is established [11].

## Conclusions

The presentation of an incarcerated Bochdalek hernia in adults is very rare. However, early diagnosis and timely surgical management are crucial aspects of the patient's prognosis.

This report enriches the medical literature by demonstrating that when carrying out the diagnostic approach to a patient with abdominal pain and intestinal obstruction, we must have multiple differential diagnoses in mind even if they are rare clinical entities.

In retrospect, the viability of the transverse and ascending colon in our case was not favorable and was the reason for performing an extended right hemicolectomy. The surgeon determined in each case whether resection or conservation of the herniated tissues was necessary, as well as the possibility of performing an anastomosis or a stoma.

It should be emphasized that the neurological status, oxygen saturation level, heart rate, respiratory rate, urinary index, and intra-abdominal pressure must always be closely monitored since any changes in these parameters may be early warning signs of requiring for a new emergency intervention.
